# Do Daily Compensatory Health Beliefs Predict Intention to Quit and Smoking Behavior? A Daily Diary Study during Smoking Cessation

**DOI:** 10.3390/ijerph17176419

**Published:** 2020-09-03

**Authors:** Melanie A. Amrein, Janina Lüscher, Corina Berli, Theda Radtke, Urte Scholz

**Affiliations:** 1 Department of Psychology, University of Zurich, Binzmuehlestrasse 14, 8050 Zurich, Switzerland; janina.luescher@psychologie.uzh.ch (J.L.); corina.berli@psychologie.uzh.ch (C.B.); urte.scholz@uzh.ch (U.S.); 2 School of Psychology and Psychotherapy, Witten/Herdecke University; Alfred-Herrhausen-Straße 45, 58455 Witten, Germany; Theda.Radtke@uni-wh.de

**Keywords:** compensatory health beliefs, smoking cessation, intention, ecological momentary assessment

## Abstract

Compensatory health beliefs (CHBs) are a means to cope with motivational conflicts between intended health goals and the temptation for an unhealthy behavior. As CHBs can fluctuate on a daily basis, this study examined how daily CHBs are associated with daily intention to quit smoking and daily number of cigarettes smoked before and after a quit date at the between- and within-person level. The study comprised a prospective longitudinal design and investigated 83 women and 83 men for 32 consecutive days during an ongoing joint self-set quit attempt. Daily CHBs varied from day to day and between individuals. At the between-person level, higher women’s mean CHBs were associated with lower intention (*b* = −0.23, *p* = 0.04) and at the 10% level with more cigarettes smoked after the quit date (rate ratio (RR) = 1.92, *p* = 0.07). At the within-person level, women’s higher than usual CHBs were unrelated to intention to quit, but were related to less smoking before (RR = 0.96, *p* = 0.03) and at the 10% level after the quit date (RR = 0.91, *p* = 0.09). A marginally positive association between daily CHBs and smoking at the within-person level emerged for men. The negative effect of daily CHBs at the between-person level on smoking seems to unfold after the quit attempt and for women only.

## 1. Introduction

People can apply different strategies that allow them to behave unhealthily such as smoking cigarettes without having a guilty conscience or feeling regrets. In order to counter such upcoming unpleasant feelings or cognitive dissonance [1], the perceived risk of an unhealthy behavior can be neutralized by adopting specific beliefs [2], such as compensatory health beliefs (CHBs; [3]. CHBs are beliefs that an unhealthy, but simultaneously desirable behavior can be compensated for by engaging in another healthy behavior that has a positive effect on health. Consequently, an individual would think that being physically active can compensate the negative effects of smoking cigarettes on health (cf. [3]). In the context of smoking behavior, previous studies indicate that people with higher CHBs smoke more cigarettes [4] and have lower intention to quit smoking [5]. As not only quitting smoking, but also the reduction of daily cigarette intake can have tremendous health benefits [6], the association between CHBs and smoking behavior changes should be further investigated, and thus is the focus of the present study.

According to the compensatory health beliefs model [7], CHBs are relevant in moments of motivational conflicts to justify smoking a cigarette. A motivational conflict can result when the desire to engage in a tempting behavior is in contrast with a health goal. The model also proposes that especially when people have a high motivation to follow a health goal (e.g., not to smoke) and experience a desire (e.g., the desire to smoke), CHBs will be activated as a way to cope with the situation [7]. After a quit date, smokers are highly motivated not to smoke, because, for example, they decided smoking is not worth the increased risk of fatal diseases such as stroke or cancer [8,9]. At the same time, especially during the first days after a quit date, smokers feel strong urges for cigarettes [10]. We assume that especially on days after a quit date, when people are highly motivated to stop smoking, CHBs are relevant for smoking behavior compared with days when people do not attempt to quit smoking. To date, no study has analyzed the role of CHBs during smoking cessation around a self-set quit date.

Previous studies have investigated the relation between CHBs and smoking only at the between-person level with mainly cross-sectional designs [4,5,11]. This can, for example, answer the question of whether people with higher CHBs smoke more cigarettes than people with lower CHBs. These kinds of analyses, however, do not tell us anything about associations between CHBs and the behavior of individuals over time [12]. There is evidence that CHBs can vary from day to day and are relevant for unhealthy eating [13]. However, no study to date has investigated the role of daily CHBs in smoking behavior change. Examining associations between CHBs and smoking on a daily level within individuals can, for example, answer the question of whether on days with higher CHBs than usual a person smokes more cigarettes than on days with lower CHBs than usual. To avoid ecological fallacy, thinking that relationships observed for a group necessarily hold for an individual, it is essential to assess the relation between CHBs and smoking behavior at the intraindividual level with ecological momentary assessments to assess experiences in real-time, in real-world settings, over time and across contexts [14]. Therefore, it is essential to disentangle the between- from the within-person component of CHBs to assess how daily CHBs are related to health behaviors at the between- and at the within-person level [12]. With an intensive longitudinal study design, we aim to investigate between-person associations with mean CHBs across the study period and within-person associations with a daily fluctuation of CHBs shortly after a self-set quit date compared with the days before.

### Aim of the Present Study

The aim of the present study was to test whether daily CHBs are associated with the daily intention to quit and daily number of cigarettes smoked during smoking cessation. We had the following theory-driven hypotheses: (1) In line with previous findings [11], we assumed that smokers with higher mean CHBs have a lower intention to quit than smokers with lower mean CHBs at the between-person level (H1a). As CHBs are used in moments of motivational conflicts that in turn are assumed to be highly relevant in health behavior change [7], we assumed that the effect of CHBs on intention at the between-person level is stronger for the time of the quit date and the days after compared with the days before (H1b). As CHBs can also vary from day to day [13], we hypothesized that on days with higher CHBs than usual, smokers report a lower intention to quit (H1c). Again, this effect of daily CHBs on intention to quit at the within-person level is assumed to be stronger for the time of the quit date and the days after compared with the days before (H1d). (2) In line with previous findings [4], we assumed that smokers with higher mean CHBs smoke more cigarettes compared to women and men with lower mean CHBs at the between-person level (H2a). Again, we assumed that this effect of CHBs on the number of cigarettes smoked at the between-person level is stronger for the time of the quit date and the days after compared with the days before (H2b). To investigate the role of daily CHBs at the within-person level, we hypothesized that on days with higher CHBs than usual, smokers smoke more cigarettes (H2c) and that the associations are stronger for the time of the quit date and the days after compared with the days before (H2d).

## 2. Method

### 2.1. Procedure and Participants

This study was part of a larger project “Individual regulation and dyadic exchanges during an on-going quit attempt in dual smoker couples” (for a detailed description see [15,16]) and was funded by the Swiss National Science Foundation (PP00P1_133632/1). The project was approved by the ethics committee of the University of Bern’s Faculty of Human Sciences in Switzerland (2011-11-14409).

The study comprised a prospective longitudinal design and investigated heterosexual adult dual-smoker couples for 32 consecutive days during an ongoing joint self-set quit attempt. Couples were recruited via newspapers, web pages, public advertising, bulletins, and a market research institution. Couples received CHF 100 (approx. USD 107) for their participation in the study when completing the daily diaries and participating in the follow-up approximately one month after the joint self-set quit date.

Eligibility criteria included smoking at least one cigarette daily, intending to quit smoking together with their partner, being in a committed relationship for at least one year and living together for at least six months. Participants were excluded if they attended a professional program for smoking cessation, worked in shift work, did not speak German or if the woman was pregnant. Participants were invited to a baseline assessment where they provided sociodemographic data and signed an informed consent. Further, they were instructed to define a joint self-set quit date, but the participants did not receive any treatments that supported them to quit smoking as part of the study. Ten days before this joint self-set quit date, the assessment of smoking behavior, intention to quit, CHBs and other smoking-relevant constructs started with a daily evening diary using study-provided smartphones and lasted until 21 days after the quit date.

At baseline, 85 dual-smoker couples participated in the study. Two couples dropped out before the quit date and were excluded from further analyses. As the focus was not on couple effects, women and men were analyzed separately. The final data included 83 women and 83 men with 32 data points from the daily evening diary each. Of in total 2656 possible diary entries, the numbers of completed diary entries for women (*n*_Women_ = 2313) and men (*n*_Men_ = 2207) were statistically not different, χ^2^ = 1.34, *p* = 0.247. The mean age of women (M_Women_ = 38.5, SD_Women_ = 14.6) and men (M_Men_ = 40.7, SD_Men_ = 14.5) did not differ (*t*(162) = −0.95, *p* = 0.345). Furthermore, 32 (37.7%) women went to high school, 40 (47.1%) women to secondary school and 9 (10.6%) women only attended primary school. For men, 24 (28.2%) went to high school, 45 (52.9%) to secondary school and 14 (16.5%) to primary school. Graduation did not differ for women and men (χ^2^ = 2.49, *p* = 0.287). Lastly, 52 (61.2%) women and 63 (74%) men were employed, 9 (10.6%) women and 9 (10.6%) men were retired and 3 (3.5%) women and 2 (2.4%) men were unemployed. Working status did also not differ for women and men (χ^2^ = 0.53, *p* = 0.767).

### 2.2. Measures

All items were presented in German during the 32 diary days. Table 1 gives an overview of means, standard deviations, mean difference statistics and effect size (Cohen’s *d*) of all main measures of this study.

Daily number of cigarettes smoked was assessed by the item “Did you smoke today (including only one puff)?” The response format was no (0) or yes (1). If the response was yes, participants were asked to report how many cigarettes they had smoked [17]. If smokers had not smoked any cigarette, the number of cigarettes smoked was coded as zero. If participants indicated to smoke only a part of a cigarette (e.g., 1.5), this was rounded up to the next integer (in this case 2).

Daily intention to quit was assessed with the item “I have the intention to refrain from smoking tomorrow.” [18]. The response format ranged from (1) “today not at all true” to (6) “today completely true”.

Daily compensatory health beliefs were assessed on a daily basis with a smoking-specific CHB item adapted from Knäuper et al. [3] “I can compensate my smoking behavior with other healthy behaviors, e.g., working out, healthy eating.” (cf. [5]). The response format ranged from (1) “today not at all true” to (6) “today completely true”.

### 2.3. Data Analysis

As we were interested in the within- and between-person associations between daily CHBs, daily intention to quit smoking and daily number of cigarettes smoked, linear mixed models were applied in R 3.4.1 [19]. This allowed modelling of the variation of coefficients between different time points for individuals at the within-person level and between individuals at the between-person level [20].

To analyze the association between daily CHBs and daily intention, we applied a linear mixed model in the lme4 package with the lmer function in R. Daily number of cigarettes smoked is a count variable. To analyze the association between daily CHBs and daily smoking, we thus applied a generalized linear mixed negative binomial model [21] with a logarithmic link function using the glmmadmb function in the glmmADMB package in R. The negative binomial model has the advantage that mean and variance can differ from each other [22]; thus, a better model fit should be achieved. For the negative binomial model, rate ratios (RRs) were calculated. RRs are interpreted as the percentage increase (values > 1) or decrease (values < 1) in the outcome variable (daily number of cigarettes smoked) for one unit increase in the predictor (e.g., CHBs; [21]). Both models were analyzed for women and men separately, as the focus was not on couple effects.

To examine the amount of variability at both levels, intra-class correlations (ICCs) were calculated for daily CHBs, daily intention to quit and daily number of cigarettes smoked (see Table 1). The ICC indicates the amount of between-person variance in relation to total variance (see [23]). Effect sizes were calculated with Cohen’s *d* for correlated sample comparisons [24].

First, a grand-mean-centered between-person variable for CHB was generated to assess whether the overall score for CHBs for each participant is related to intention and daily number of cigarettes smoked. To obtain the mean CHB variable, the grand mean (mean score across subjects and time points) was subtracted from the mean score of each individual. Second, a within-person variable for CHBs (CHBs-within) was generated (see [20]). Here, the mean score of each individual was subtracted from the daily raw scores of each individual. Thus, the CHBs-within variable indicates the deviation of the daily CHB value from the personal CHB mean score over time.

Each model contained the following predictors: A linear time variable (0 for the first diary day; 31 for the last diary day), the within-person variable of CHBs (CHBs-within), the grand-mean centered between-person variable of CHBs (mean CHBs) and the binary variable quit date (0 = days before the quit date; 1 = the day of the quit date and the days after). To test whether the quit date moderates the associations between the main variables, interaction terms between the predictors and the quit date indicator were entered in the model. Simple slopes analyses were computed to compare associations before and after the quit date [25]. In addition, we specified a maximal random effects structure for all models [26] including random slopes for variables at the within-person level. The R packages used to analyze the models are not testing for significance of the random effects.

## 3. Results

Intra-class correlations (ICCs) of all main variables are displayed in Table 1 and ranged from 0.15 to 0.64. For both women and men, ICCs for intention to quit (0.15–0.18) and for daily number of cigarettes smoked (0.28–0.39) were small, indicating that the proportion of the total variance that is due to between-person differences was rather low. The ICC for daily CHBs ranged from 0.48–0.64. An ICC of 0.48 indicates that half of the total variance in CHBs was due to stable between-person differences.

At the quit date and the days after, women and men showed higher intention to quit and smoked less cigarettes (see Table 1). For women, the mean score of daily CHBs was marginally significantly higher at the quit date and the days after (*M* = 3.03, *SD* = 1.17) compared with the days before (*M* = 2.80, *SD* = 1.12, *t*(82) = −1.98, *p* = 0.052, *d* = 0.19). After the quit date, 27 of 83 women and 23 of 83 men smoked no cigarettes at all.

### 3.1. Daily CHBs Predicting Daily Intention to Quit

**Figure 1:**
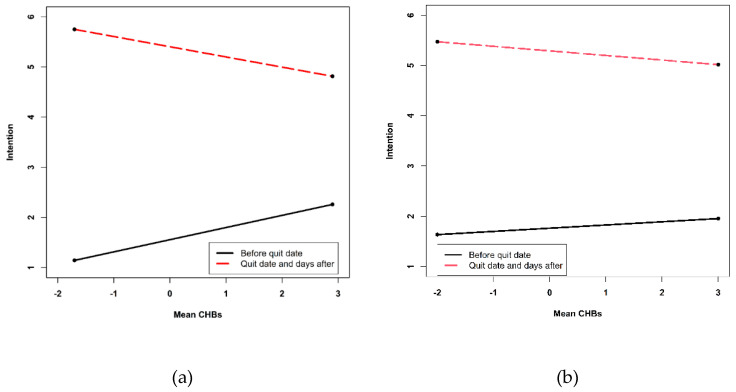
Simple slopes of the between-person variable of daily CHBs (mean CHBs) predicting daily intention to quit smoking before and after quit date for women (**a**) and men (**b**). The between-person variable of daily CHBs is centered so that 0 is indicating the mean CHB value of the average women.

The results of the models testing daily CHBs as a predictor for intention to quit for women and men are presented in Table 2. The intercept of 1.70 for women and 1.76 for men on a scale from 1 to 6 shows the estimated value for intention on the first diary day for the average women or men when all covariates are equal to zero. Women reported 3.58 and men 3.52 units higher intention at the quit date and the days after compared with the days before (*p* < 0.001) on a range from 1 to 6. For both sexes, the time slope after the quit date (*b*_Women_ = 0.19, *p*_Women_ < 0.001; *b*_Men_ = 0.17, *p*_Men_ < 0.001) differed from the time slope before the quit date (*b*_Women_ = −0.02, *p*_Women_ < 0.001; *b*_Men_ = −0.02, *p*_Men_ = 0.002), indicated by significant negative interaction terms (*b*_Women_ = −0.21, *p*_Women_ < 0.001; *b*_Men_ = −0.19, *p*_Men_ < 0.001). In line with H1a but only for women after the quit date, there was a negative effect for mean CHBs (*b* = −0.23, *p* = 0.038) on intention. This means that after the quit date, women with higher mean CHBs reported lower intention to quit compared to women with lower mean CHBs. This association was different compared to the days before the quit date, indicated by a significant interaction term (*b* = −0.51, *p* = 0.002). However, not in line with H1b, the association between mean CHBs and intention before the quit date was positive (*b* = 0.28, *p* = 0.006). Figure 1 illustrates the interaction effects of mean CHBs and the quit date on intention to quit for women and men. No significant effect at the between-person level was found for men.

At the within-person level, a marginally positive association between daily CHBs and intention at the within-person level emerged for men before (*b* = 0.09, *p* = 0.076) and after the quit date (*b* = 0.06, *p* = 0.078), resulting in no significant interaction term (*b* = −0.04, *p* = 0.540). This indicates that, in contrast with H1c and H1d, daily fluctuations in CHBs around the person mean were not negatively associated with intention before and after the quit date. The random effect for the quit date showed the highest variability compared to the other random effects in the models predicting intention for both women and men.

### 3.2. Daily CHBs Predicting Daily Number of Cigarettes Smoked

**Figure 2:**
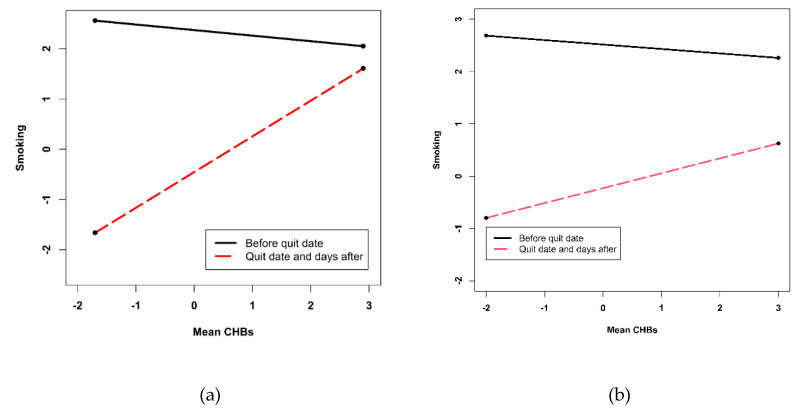
Simple slopes of the between-person variable of daily CHBs (mean CHBs) predicting daily number of cigarettes smoked before and after quit date for women (**a**) and men (**b**). The between-person variable of daily CHBs is centered so that 0 is indicating the mean CHBs value of the average women.

The results of the models testing daily CHBs as a predictor for daily number of cigarettes smoked for women and men are presented in Table 3. The intercept RR provides the estimated number of cigarettes smoked on the first diary day when all covariates are equal to zero: RR_Women_ = 10.53 and RR_Men_ = 12.40. At the quit date and the days after, women smoked on average 93% less cigarettes compared with the days before the quit date (RR = 0.07, *p* < 0.001). Men smoked 94% less cigarettes (RR = 0.06, *p* < 0.001) at the quit date and the days after compared with the days before the quit date. No linear time trend was found before and after the quit date for both sexes. In line with H2a but only for women, higher mean CHBs were marginally positively related with smoking (RR = 1.92, *p* = 0.071) at the quit date and the days after, indicating an increase of 92% more cigarettes smoked. Before the quit date, no association emerged for women (H2b), resulting in a significant interaction term. Figure 2 illustrates this significant interaction effect of mean CHBs and the quit date on the number of cigarettes smoked for women and men. No significant effect at the between-person level was found for men. This indicates that, in contrast with H2a and H2b, men with higher mean CHBs did not smoke more cigarettes before and after the quit date.

At the within-person level for women only, daily CHBs were negatively related with the daily number of cigarettes before (RR = 0.96, *p* = 0.026) and marginally negatively related after the quit date (RR = 0.91, *p* = 0.092), resulting in no significant interaction term between daily CHBs and quit date. This indicates that, in contrast with H2c, on days with higher daily CHBs than usual, women smoked on average 4% less cigarettes before, and 9% less cigarettes after the quit date. No effects at the within-person level was found for men. Again, the random effect for the quit date showed the highest variability compared to the other random effects in the models predicting smoking for both women and men.

## 4. Discussion

This is the first study examining the role of daily CHBs during smoking cessation around a self-set quit date using a daily diary design. We found in parts support for our assumption that daily CHBs play an important role for the daily intention to quit and daily number of cigarettes smoked at a quit date and the days after compared with the days before.

The results showed that at the quit date and the days after, female smokers reported higher daily CHBs compared with the days before, and that CHBs are less relevant for smoking before the quit date. This is in line with the assumption that CHBs are more salient after a quit date because smokers may experience more situations of motivational conflicts and use CHBs as justifications for unhealthy behavior [7]. Thus, when investigating the role of CHBs in smoking behavior, it seems essential to consider whether people attempt to quit smoking as a possible moderator.

At the between-person level, at the quit date and the days after, women with higher mean CHBs reported lower intention to quit smoking compared to women with lower mean CHBs (H1a). In contrast, before the quit date, an unexpected reversed association emerged (H1b). Higher values of mean CHBs at the between-person level were related with higher intention to quit. This is not in line with our theoretical assumption that CHBs are negatively related with the intention to behave healthily for a particular behavior (c.f. [11,27]]). A main difference compared to previous studies is that in the present study, smokers had already defined a quit date. Thus, future studies investigating the relation between CHBs and the intention to quit smoking could directly compare people not planning to stop smoking with people who already defined a quit date at a later point in time. This would allow for the question of how the awareness of a self-set quit date might change the relation between CHBs and the intention for smoking behavior to be answered. Furthermore, no associations between daily CHBs and the intention to quit smoking emerged at the within-person level for women (H1c). For men, higher CHBs than usual were marginally associated with higher intention on the same day. Further investigations on the temporal effects of daily CHBs on health behavior change are needed. Ideally, this should be combined with developing theoretical assumptions on the temporal dynamics of daily CHBs, daily intentions, and daily behavior (cf. [28]).

At the quit date and the days after, women with higher mean CHBs smoked marginally more cigarettes than women reporting lower mean CHBs (H2a). The quit date moderated the association between mean CHBs and daily number of cigarette smoked at the between-person level, again only for women (H2b). Contrarily to our hypothesis at the within-person level, women smoked less cigarettes on days with higher CHBs than usual before and after the quit date (H2c). Interestingly, Radtke et al. [11] reported also a negative relation between CHBs and smoking, even though it was not significant. One possible explanation is that on days when people do not experience smoking urges or situations of motivational conflicts, they smoke no or less cigarettes, but at the same time, can still have a strong belief that smoking can be compensated for by engaging in another healthy behavior. To further enlighten this point, future studies should also assess people’s smoking urges or motivational conflicts when investigating the role of CHBs in smoking behavior.

This was the first study analyzing CHBs at a daily basis to investigate whether on days with higher daily CHBs than usual people smoke more cigarettes and have lower intention to quit smoking on these days. Not in line with our hypotheses, no negative associations at the within-person level between CHBs and intention (H1c) and no positive associations between CHBs and smoking were found (H2c). One reason might be that the CHB item (“I can compensate my smoking behavior with other healthy behaviors, e.g., working out, healthy eating.” based on [11]) was formulated quite generally and thus was unrelated to a certain time reference, such as today. People could have answered this item as trait CHBs that address the overall belief of CHBs. However, the ICC of CHBs in this study was 0.48, indicating that CHBs also varied among people from day to day. As no validated CHB scale exist to date, future studies may want to distinguish more explicitly between trait CHBs, which are stable over time, and state CHBs, which are situations specific and are dependent on daily variation of CHBs [29]. To investigate CHBs at the within-person level, items asking about CHBs should be formulated as state CHBs that clearly refer to a specific moment, e.g., “At the very moment of smoking a cigarette, I was thinking that it is OK to smoke this cigarette as long as I smoke less the next day.” (cf. [5]). Such a formulation would directly address the act of an unhealthy behavior, in this case smoking. This goes in line with the assumption that the overall belief of CHBs can be distinguished from compensatory health behavior (cf. [30]). Compensatory health behavior is defined as the behavior an individual actually engages in to compensate for the negative health effects of another unhealthy behavior. There is evidence that CHBs and compensatory health behavior result in different associations with health behavior change intention and health behavior [30]. In the present study, it is not possible to make assumptions regarding whether people actually compensated the unhealthy behavior later on. In sum, future studies examining within-person processes of CHBs on a daily basis should distinguish between overall beliefs of CHBs and CHBs that arise in specific situations to justify the unhealthy behavior.

Interestingly, different relations emerged for men compared to women. None of the existing studies investigating smoking behavior analyzed specific gender differences regarding the effect of CHBs [4,5,11]. In this study, although women and men reported equivalent beliefs of compensating the own smoking behavior with other health behaviors, women seemed to apply these beliefs to justify their smoking behavior, resulting in less intention to quit smoking and in more cigarettes smoked at the quit date and the days after. When applying CHBs to justify smoking behavior, situations of motivational conflicts that arise from the interplay between affective states (e.g., cravings) and health goals have to occur [7]. One explanation may be that women and men differ from each other in their perception of the discrepancy among these cognitions. For example, women appear to experience greater subjective stress, craving, arousal and negative emotion in response to stress cues [31]. As mentioned above, strong craving for cigarettes in stressful situations represents a key factor for the formation of motivational conflicts that in turn lead to the activation of CHBs to justify smoking behavior. Thus, in this study, women may have experienced more situations of motivational conflicts due to greater subjective stress and craving. Future longitudinal studies should explicitly measure the number of motivational conflicts people experience to investigate this gender effect of the relationship between CHBs and indicators of behavior change.

### Limitations and Outlook

One limitation of this study is that smoking was assessed via self-report, but additional objective measurements such as biochemical assessments may improve accuracy [32]. Especially during quit attempts, smokers often misclassify themselves as non-smokers [33]. In this study, a carbon monoxide (CO) test of expired air was conducted with the Smokerlyzer (Bedfont Instruments, Harrietsham, UK) to measure point prevalence of abstinence, but only at follow-up and not during the diary phase (see [15]). It is recommended to combine self-report measures and objective measures at the same time [34]. One innovative objective measure in smoking cessation research is a mobile phone-based breath carbon monoxide meter that is easy to handle for participants [35]. A further limitation is that the results cannot be generalized for smokers who are not in a committed romantic relationship with another smoking person. Another limitation is that, due to the correlational nature of this study, no causal inferences can be drawn. However, studies using experimental manipulations regarding CHBs, for example, in the form of a controlled laboratory experiment, are still lacking. Future studies might want to apply such strong designs to test the effect of CHBs on smoking in smoking cessation to allow for causal inferences.

An important implication from the presented finding is that CHBs could serve as an approach for interventions to reduce smoking behavior. In detail, future studies could target reducing the negative effects of CHBs on smoking, especially after people decided to stop smoking. As proposed by Rabiau et al. [7], one way to intervene is at the stage of CHB activation, e.g., raising the awareness of the negative effects of CHBs for health to prevent people using CHBs as justification. However, as CHBs seem to be relevant for emotion regulation in smokers, interventions may strengthen people’s ability to cope with conflict situations, e.g., focus on relevant constructs such as self-efficacy [36]. Whether or not such interventions targeting CHBs are indeed effective in improving smoking behavior or abstinence needs to be investigated in future studies.

## 5. Conclusions

Our study contributes to the literature on health behavior change in that it is the first intensive longitudinal study exploring the association between daily compensatory health beliefs, daily intention to quit and daily smoking behavior in people’s everyday lives during smoking cessation. The belief that smoking can be compensated for by engaging in another healthy behavior varied from day to day, but only associations at the between-person level for women were confirmed. In contrast, at the within-person level for women, a negative relation between daily CHBs and smoking appeared. Future studies should further investigate how daily fluctuations of compensatory health beliefs can effect unhealthy behaviors.

## Figures and Tables

**Table 1:** Means, standard deviations, *t*-test comparing 10 days before the quit date and the quit date and 21 days after, effect sizes and intra-class correlations (ICC) of main variables displayed for women and men.

	10 Days before Quit Date		Quit Date and 21 Days after					
Variables	M	SD	M	SD	*t* Value	*p*	Cohen’s *d*	ICC
**Women**								
1. CHBs	2.80	1.12	3.03	1.17	−1.98	0.052	0.19	0.48
2. Intention	2.55	1.02	4.82	1.17	−12.24	<0.001	2.07	0.18
3. Cig.	12.39	6.28	4.31	5.87	12.06	<0.001	1.32	0.39
**Men**								
1. CHBs	2.69	1.17	2.72	1.29	−0.48	0.634	0.03	0.64
2. Intention	2.50	1.13	4.87	1.03	−13.86	<0.001	2.20	0.15
3. Cig.	14.26	7.93	4.45	5.17	11.87	<0.001	1.42	0.28

Note: *N*_Men_ = 81; *N*_Women_ = 82; ICC = Intra-class correlations; CHBs = Compensatory health beliefs; Cig. = daily number of cigarettes smoked.

**Table 2:** Linear mixed model of daily intention to quit smoking regressed on daily CHBs and interaction with quit date for women and men.

	**Women**			**Men**		
**Fixed Effects**	**Estimate (SE)**	**95% CI**	**Estimate Post Quit Date ^a^ (SE)**	**Estimate (SE)**	**95% CI**	**Estimate Post Quit Date ^a^ (SE)**
Intercept	1.70 *** (0.12)	(1.50, 1.90)		1.76 *** (0.14)	(1.53, 1.99)	
Quit	3.58 *** (0.18)	(3.28, 3.88)		3.52 *** (0.19)	(3.21, 3.84)	
Time	0.19 *** (0.01)	(0.17, 0.21)		0.17 *** (0.01)	(0.15, 0.19)	
Time by Quit	−0.21 *** (0.01)	(−0.23, −0.19)	−0.02 *** (0.01)	−0.19 *** (0.01)	(−0.21, −0.17)	−0.02 ** (0.01)
CHBs_between-person_	0.28 ** (0.10)	(0.12, 0.44)		0.05 (0.11)	(−0.12, 0.22)	
CHBs_between-person_ by quit	−0.51 ** (0.16)	(−0.77, −0.25)	−0.23 * (0.11)	−0.15 (0.14)	(−0.37, 0.08)	−0.10 (0.09)
CHBs_within-person_	0.02 (0.04)	(−0.05, 0.09)		0.09 ^#^ (0.05)	(0.01, 0.18)	
CHBs_within-person_ by quit	0.01 (0.05)	(−0.07, 0.09)	0.03 (0.03)	−0.04 (0.06)	(−0.13, 0.06)	0.06 ^#^ (0.03)
**Random Effects**	**Variance (SE)**			**Variance (SE)**		
Intercept	0.87 (0.93)			1.29 (1.14)		
Time	0.01 (0.05)			0.01 (0.05)		
Quit	1.89 (1.37)			2.16 (1.47)		
CHBs_within-person_	0.05 (0.21)			0.07 (0.26)		
CHBs_within-person_ by quit	0.06 (0.24)			0.05 (0.22)		
Residual	0.70 (0.83)			0.77 (0.88)		

Note: *N*_Men_ = 83; *N*_Women_ = 81; ^#^ = *p* < 0.01/* = *p* < 0.05/** = *p* < 0.01/*** = *p* < 0.001; Estimate = unstandardized regression coefficients; SE = standard errors; 95% CI = 95% confidence interval; ^a^ Simple slope estimate for post quit date; Quit = quit date (0 = 10 days before quit date; 1 = quit date and 21 days after quit date); Time = 32 diary days (0 = first diary day, 31 = last diary day).

**Table 3:** Generalized linear mixed negative binomial model of daily smoking regressed on daily CHBs and on interaction with quit date for women and men.

	**Women**				**Men**			
**Fixed Effects**	**Estimate (SE)**	**95% CI**	**RR**	**Estimate Post Quit Date ^a^ (SE)**	**Estimate (SE)**	**95% CI**	**RR**	**Estimate Post Quit Date ^a^ (SE)**
Intercept	2.35 *** (0.07)	(2.24, 2.46)	10.53		2.52 *** (0.07)	(2.40, 2.63)	12.40	
Quit	−2.71*** (0.32)	(−3.23, −2.18)	0.07		−2.78 *** (0.29)	(−3.26, −2.30)	0.06	
Time	−0.01 (0.01)	(−0.01, 0.01)	1.00		−0.01 (0.01)	(−0.02, 0.01)	0.99	
Time by quit	−0.01 (0.01)	(−0.02, 0.00)	0.99	−0.01 (0.01)	0.01 * (0.01)	(0.00, 0.02)	1.01	0.01 (0.23)
CHBs_between-person_	−0.10 (0.06)	(−0.21, 0.00)	0.90		−0.09 (0.06)	(−0.18, 0.01)	0.92	
CHBs_between-person_ by quit	0.76 * (0.30)	(0.27, 1.24)	2.13	0.65 ^#^ (0.36)	0.36 (0.23)	(−0.02, 0.74)	1.43	0.27 (0.29)
CHBs_within-person_	−0.04 *(0.02)	(−0.07, −0.01)	0.96		−0.02 (0.02)	(−0.04, 0.01)	0.99	
CHBs_within-person_ by quit	−0.04 (0.04)	(−0.10, 0.02)	0.96	−0.09 ^#^ (0.05)	−0.02 (0.05)	(−0.10, 0.05)	0.98	−0.04 (0.06)
**Random Effects**	**Variance (SE)**				**Variance (SE)**			
Intercept	0.31 (0.56)				0.33 (0.58)			
Quit	6.36 (2.52)				5.69 (2.39)			
Time	0.01 (0.05)				0.01 (0.06)			
CHBs_within-person_	0.01 (0.09)				0.01 (0.06)			
CHBs_within-person_ by quit	0.03 (0.16)				0.05 (0.21)			

Note: *N*_Men_ = 83; *N*_Women_ = 81; ^#^ = *p* < 0.01/* = *p* < 0.05; ** = *p* < 0.01; *** = *p* < 0.001; Estimate = unstandardized regression coefficients; SE = standard errors; 95% CI = 95% confidence interval; *RR* = Rate ratios; ^a^ Simple slope estimate for post quit date; Quit = quit date (0 = 10 days before quit date; 1 = quit date and 21 days after quit date); Time = 32 diary days (0 = first diary day, 31 = last diary day).
